# Development of a FACS-based Smac/DIABLO release assay to monitor individual drug responses and to pave the way towards patient-tailored therapies

**DOI:** 10.1186/1476-9255-12-S1-P5

**Published:** 2015-04-16

**Authors:** Jigyasa Arora, Ralf Michael Zwacka, Andrea Mohr

**Affiliations:** 1 School of Biological Sciences, University of Essex, Wivenhoe Park, Colchester, Essex, CO4 3SQ, UK

## 

The induction of programmed cell death (apoptosis) in tumour cells is an essential cellular and molecular process in response to cancer treatment, and important for its success. Resistance to such therapies can occur at different molecular levels. One essential step in apoptosis signalling is the release of several factors from mitochondria into the cytosol. In this context, Smac/DIABLO, aside from cytochrome c, has been recognised as one of these key pro-apoptotic factors. Smac/DIABLO binds to the anti-apoptotic factor XIAP in the cytosol thereby blocking its capacity to inhibit activation of caspases. As XIAP is known to be up-regulated in many cancer cells leading to treatment resistance, it is important to be able to monitor drug responses on an individual and single cell level in primary tumour material. To this end, we developed a FACS-based Smac/DIABLO release assay. Using the cytotoxic drug 5-Fluorouracil, we could show that the signal for Smac/DIABLO increased, reflecting the appearance of Smac/DIABLO in the cytosol. This signal shift could be inhibited by addition of the pan-caspase inhibitor zVAD. Moreover, these results correspond to data from biochemical analysis of mitochondrial and cytosolic fractions that also show a release of Smac/DIABLO into the cytosol. Thus, this FACS method represents a generally useful tool to understand apoptosis signalling and resistance in cancer cells, which can inform treatment options for individual patients.

